# Deciphering Stress Resilience in Black Pepper (*Piper nigrum* L.): From Current Advances to Emerging Opportunities

**DOI:** 10.3390/ijms27146458

**Published:** 2026-07-21

**Authors:** Acga Cheng, Kho Pei Ee

**Affiliations:** 1 Institute of Biological Sciences, Faculty of Science, Universiti Malaya, Kuala Lumpur 50603, Malaysia; khope@mpb.gov.my; 2 Malaysian Pepper Board, Kuching 93450, Malaysia

**Keywords:** artificial intelligence, abiotic stressors, biotic stressors, black pepper, genetic improvement, rhizosphere, sustainable agriculture

## Abstract

Black pepper (*Piper nigrum* Linn.), one of the world’s most economically important spice crops, is increasingly challenged by climate-related stresses, emerging pests and diseases, and declining soil health, all of which threaten its productivity and sustainability. While previous reviews have predominantly focused on black pepper genomic resources, breeding strategies, and disease management, the integration of multi-omics technologies, microbiome science, and artificial intelligence (AI) to enhance its stress resilience has received comparatively limited attention. This review synthesizes recent advances in the molecular mechanisms underlying black pepper responses to biotic and abiotic stresses, with emphasis on omics approaches (such as genomics and transcriptomics), as well as the roles of beneficial microbial communities in enhancing stress tolerance, nutrient acquisition, and disease suppression. We further discuss emerging microbiome-assisted strategies, including the development of beneficial microbial consortia and targeted manipulation of microbial functions, for enhancing black pepper resilience under changing environmental conditions. In addition, we explore how AI-driven analytical approaches can integrate complex multi-omics and microbiome datasets to unravel the complex molecular networks governing black pepper–microbe interactions under stress conditions and accelerate precision breeding. By integrating genomics, microbial ecology, and AI, this review presents a systems-level framework for understanding and improving stress resilience in black pepper. This interdisciplinary perspective highlights new opportunities to accelerate the development of climate-resilient cultivars and advance sustainable black pepper production.

## 1. Introduction

Black pepper (*Piper nigrum*; 2*n* = 52), a perennial vine belonging to the Piperaceae family and widely recognized as the “King of Spices”, is cultivated extensively throughout tropical regions. It serves as a major source of income and livelihood for millions of smallholder farmers [1]. However, black pepper production is increasingly threatened by climate change, environmental degradation, emerging pests and pathogens, and the simultaneous occurrence of multiple abiotic and biotic stresses that collectively compromise crop productivity and sustainability [2]. In natural and agricultural ecosystems, crops in tropical regions are frequently exposed to concurrent drought, heat, nutrient limitation, and/or disease pressure, resulting in stress responses that cannot be fully explained by single-factor studies. Consequently, our understanding of the mechanisms that underpin crop resilience under realistic field conditions remains limited, creating a significant knowledge gap for many economically important crops [3], including black pepper.

To address this challenge, recent advances in plant science have shifted the focus of crop adaptation research from a gene-centric model toward a systems-based framework that considers the dynamic interactions among host (i.e., plant) genetics, associated microbiomes, and environmental factors. This concept, often referred to as the “holobiont”, suggests that plant resilience is not determined solely by the host genome but is co-shaped by microbial communities inhabiting roots, rhizospheres, and internal tissues [4]. Increasing evidence from major crops, including the big three cereals rice (*Oryza sativa*), maize (*Zea mays*), and wheat (*Triticum aestivum*), demonstrates that these microbial partners can enhance nutrient acquisition, improve tolerance to drought and salinity, suppress pathogens, and modulate plant stress-response pathways. Such findings have reshaped our understanding of crop adaptation by highlighting the critical role of plant–microbiome interactions in determining resilience and productivity [5,6]. However, while this “holobiont” paradigm has gained considerable attention in major and model crops, its application to spice crops remains largely unexplored. This knowledge gap presents a valuable opportunity to investigate how plant–microbiome interactions can be harnessed to enhance stress resilience and promote sustainable black pepper production.

Despite rapid advances in high-throughput sequencing, genome editing, and artificial intelligence (AI)-driven breeding, many major black pepper-producing countries (such as Indonesia, Vietnam, and Malaysia) remain constrained by limited research infrastructure, inadequate funding, and restricted access to cutting-edge technologies [7]. Hence, there is an urgent need for scalable and cost-effective strategies that can be implemented across diverse socioeconomic settings to enhance the resilience and sustainability of black pepper production. In this context, microbiome-based technologies (such as microbial inoculants) represent promising alternatives that can improve productivity, stress tolerance, and plant health without requiring extensive genomic infrastructure or advanced breeding platforms [7,8,9]. Complementing these microbiome-based approaches, advances in multi-omics technologies and AI-driven analytical tools are providing unprecedented opportunities to elucidate the complex interactions among plants, microbes, and environmental factors. However, these disciplines are often studied independently [10,11], and a comprehensive framework linking host biology, microbial ecology, and data-driven analytics for black pepper improvement has yet to emerge.

This review therefore explores stress resilience in black pepper through the integration of multi-omics, AI, and plant–microbiome interactions. Particular emphasis is placed on how advances in genomics, microbiome science, and AI can be combined to elucidate resilience mechanisms, accelerate precision breeding, and support microbiome-assisted crop improvement. By synthesizing these complementary approaches, this review proposes a systems-level framework for developing climate-resilient, productive, and sustainable black pepper production systems.

## 2. Molecular Mechanisms Underlying Stress Responses in *P. nigrum*

### 2.1. Major Abiotic and Biotic Stressors in Black Pepper

The production of black pepper is increasingly threatened by a combination of abiotic and biotic stressors that constrain productivity, development, and sustainability. The frequency and severity of these challenges are expected to intensify under changing climatic conditions, posing significant risks to global pepper production systems [1,2].

Abiotic stresses, particularly drought and heat, pose significant challenges to black pepper production by impairing growth, photosynthesis, nutrient acquisition, and yield [12,13,14]. Similarly to other plant species, these stresses trigger excessive accumulation of reactive oxygen species (ROS), resulting in oxidative damage and necessitating the activation of antioxidant and osmotic defense mechanisms [15]. A recent study conducted by Theertha et al. [15] reported that drought-tolerant black pepper genotypes maintain higher levels of proline, soluble sugars, and antioxidant enzymes such as superoxide dismutase (SOD) and catalase (CAT), thereby enhancing stress resilience and sustaining productivity under water-deficit conditions. Soil salinity and nutrient imbalances (i.e., excessive or insufficient nutrient inputs) represent additional abiotic challenges to black pepper production in many major growing countries [14,16,17]. In Vietnam, for example, the intensive use of fertilizers in black pepper cultivation has contributed to soil degradation and increased disease incidence [17].

Beyond abiotic constraints, black pepper is susceptible to a diverse range of biotic stresses caused by fungal pathogens such as *Colletotrichum gloeosporioides* (anthracnose) and *Fusarium solani* (root rot) [18,19,20], bacterial pathogens, including *Pseudomonas* spp. (bacterial spot) [21], viral pathogens such as piper yellow mottle virus (PYMoV) and cucumber mosaic virus [22,23,24], and oomycetes such as *Phytophthora capsici* (foot rot) [25]. In addition, black pepper is affected by various pests, including thrips and mealybugs [26], mites [27], and plant-parasitic nematodes such as *Meloidogyne* spp. and *Radopholus similis* [28,29]. Taken together, these pathogens and pests can severely compromise plant health and productivity, leading to substantial yield losses [30].

Collectively, these abiotic and biotic stresses highlight the need for integrated nutrient, soil, and disease management strategies to sustain black pepper production systems [15,30]. In Malaysia, Buang et al. [31] demonstrated that tending ants may indirectly facilitate the spread of PYMoV by promoting mealybug population growth and dispersal, highlighting the importance of integrated pest management approaches that consider the ecological interactions underlying virus transmission. In addition, molecular tools such as quantitative PCR (qPCR) and high-throughput sequencing approaches, including amplicon and metagenomic sequencing, can support the early detection and monitoring of pathogen diversity. These methods enable the identification of known and emerging pathogens and provide insights into pathogen community composition, thereby facilitating more targeted and sustainable disease management strategies [32].

### 2.2. Genetic Response and Resistance in Black Pepper

Recent advances in molecular research have significantly enhanced our understanding of the genetic and regulatory mechanisms underlying stress adaptation and defense responses in black pepper (Table 1). A notable milestone was the release of BlackPepKB in 2025, the first dedicated genomic database for black pepper (https://black-pepper-genomic-resource.vercel.app/, accessed on 6 June 2026)). This comprehensive and publicly accessible resource provides an integrated platform for functional genomics, enabling gene discovery, candidate gene prioritization, and molecular breeding [33].

By and large, TFs are key regulators of plant stress responses, modulating the expression of genes involved in stress signaling and adaptation [33]. In black pepper, a recent study conducted by Charles et al. [13] identified several TF families, including WRKY, NAC, ERF (Ethylene-Responsive Factor), and MYB (Myeloblastosis) as important components of the drought-responsive regulatory network, highlighting their potential roles in enhancing stress tolerance. Several gene families and regulatory pathways have been implicated in the regulation of both abiotic and biotic stress in black pepper [43]. For example, MYB TFs have been associated with tolerance to abiotic stresses and resistance to *Phytophthora capsici*, the causal agent of foot rot disease, underscoring their potential as targets for improving overall stress resilience [30,44].

Several functional genes associated with abiotic stress tolerance have been identified in black pepper. The characteristic pungency of black pepper is primarily attributed to piperine (1-[5-(1,3-benzodioxol-5-yl)-1-oxo-2,4-pentadienyl] piperidine), and considerable progress has been made in elucidating the genes involved in its biosynthesis [37]. Beyond their role in piperine accumulation, these piperine biosynthesis genes have also been implicated in plant defense [45]. In addition, antioxidant defense-related genes, including those encoding *SOD* (superoxide dismutase), *APX* (ascorbate peroxidase), *CAT* (catalase) and *POD* (peroxidase), contribute to stress tolerance by mitigating oxidative damage through the scavenging of reactive oxygen species (ROS) generated under adverse environmental conditions [39].

Genes associated with pathogen defense have likewise attracted considerable attention due to the devastating impact of diseases on black pepper production. Resistance (R) genes are involved in the recognition of pathogen effectors and represent promising targets for breeding disease-resistant black pepper cultivars, particularly against foot rot disease caused by *Phytophthora capsici* [40]. Pathogenesis-related (PR) genes also contribute to systemic acquired resistance and enhance protection against pathogen invasion, including *P. capsici* [41]. Furthermore, genes involved in jasmonic acid-mediated defense signaling, such as *LOX* (lipoxygenase), have been implicated in resistance against fungal pathogens in black pepper [42]. Collectively, these findings provide valuable insights into the molecular mechanisms underlying stress resilience in black pepper and highlight promising genetic resources for future breeding and biotechnology-based improvement programs.

## 3. Omics and Microbiome-Integrated Genomic Strategies for Stress Resilience in *P. nigrum*

A comprehensive understanding of stress resilience in black pepper requires the integration of multiple biological layers, ranging from genetic variations to observable phenotypic traits.

### 3.1. Omics Approaches

**Figure 1:**
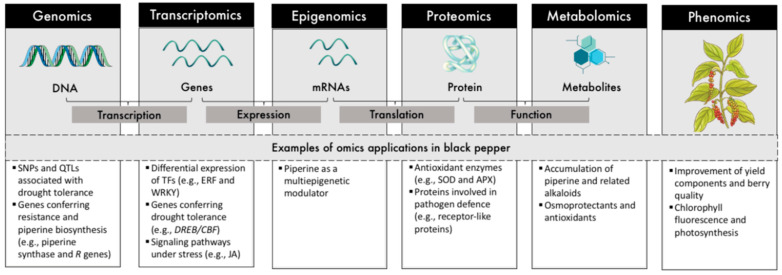
Application of omics approaches in enhancing stress resilience and yield-related components in black pepper.

Although omics-based research in black pepper remains less extensive than in major cereal crops (such as rice and maize) [46], recent advances in molecular technologies and systems biology can enhance the understanding of complex stress-response networks and facilitate the development of stress-resilient, high-yielding cultivars. Figure 1 provides an overview of omics approaches used to enhance stress resilience and yield-related components in black pepper. While presented separately for clarity, these omics layers interact extensively through complex regulatory networks involving genes, transcripts, proteins, metabolites, and phenotypic traits.

At the genomic level, advances in sequencing technologies have enabled the development of molecular markers, including single nucleotide polymorphisms (SNPs) associated with drought tolerance [12], while also facilitating the identification of stress- and resistance-related genes and quantitative trait loci (QTLs) underlying key agronomic traits in black pepper [12,47]. Complementing these studies, transcriptomic approaches have revealed dynamic changes in gene expression under stress conditions. In black pepper, several TF families, notably ERF and WRKY, have been implicated in the regulation of stress-responsive pathways [33,34,35]. Furthermore, transcriptomic analyses have highlighted the involvement of phytohormone-mediated signaling networks, particularly those associated with jasmonic acid (JA) and salicylic acid (SA), in coordinating defense responses against *P. capsici* and facilitating adaptation to environmental stresses [48]. Beyond protein-coding genes, regulatory molecules such as microRNAs (miRNAs), long non-coding RNAs (lncRNAs), and small peptides may also influence stress-responsive pathways and phenotypic outcomes, although their roles in black pepper remain largely unexplored [49].

Proteomics complements genomic and transcriptomic studies (Figure 1) by characterizing the abundance, modification, and interactions of proteins that directly mediate cellular responses to stress. In black pepper, stress-responsive proteins include antioxidant enzymes such as SOD and APX, as well as defense-related proteins involved in pathogen resistance. Variations in abundance of these proteins provide valuable insights into the molecular mechanisms underlying stress adaptation and may serve as potential biomarkers for stress tolerance [50,51]. Nonetheless, it should be noted that protein abundance does not necessarily correspond to enzyme activity, highlighting the importance of integrating proteomic data with physiological and biochemical analyses.

Metabolite profiling can reveal key physiological adaptations to environmental stress. In black pepper, metabolomic studies have primarily focused on piperine and other secondary metabolites, including phenolics and flavonoids, which contribute to both plant defense and berry quality [52,53]. Environmental stresses, especially drought, influence the accumulation of key metabolites in black pepper, including osmoprotectants and antioxidant compounds that contribute to stress tolerance by reducing oxidative damage [13]. As the principal alkaloid of black pepper, piperine has received considerable attention for its biological functions and potential roles in stress adaptation, making it an important target for future metabolomic investigations.

Epigenomics represents another important layer of regulation that influences gene expression without altering the underlying DNA sequence. Mechanisms such as DNA methylation, histone modifications, and chromatin remodeling have been shown to regulate stress responses in many crop species. Although piperine has been associated with epigenetic and transcriptional regulation in human health studies [52], epigenomic regulation in black pepper itself remains largely unexplored.

At the whole-plant level, phenomics enables the high-throughput characterization of morphological, physiological, and agronomic traits associated with stress adaptation. In black pepper, phenomic approaches have been applied to evaluate traits such as photosynthetic performance, chlorophyll fluorescence, yield components, and berry quality using imaging technologies, sensors, and automated phenotyping platforms [54]. These measurements provide an important link between molecular responses and observable plant performance under stress conditions.

The integration of multi-omics approaches with AI-driven analytical tools (such as Bayesian Networks and multi-modal deep learning) offers unprecedented opportunities to elucidate the complex molecular and physiological mechanisms underlying stress resilience, thereby enabling a systems-level understanding of stress adaptation in black pepper [33,55].

### 3.2. Microbiome-Assisted Approaches

Similarly to other crops, the rhizosphere microbiome is increasingly recognized as a key determinant of stress resilience, nutrient acquisition, and overall productivity in black pepper, highlighting its potential for sustainable crop improvement [56]. Advances in high-throughput sequencing technologies have facilitated metagenomic profiling of black pepper rhizosphere communities, enabling the identification of beneficial microbial taxa associated with plant health and disease resistance [56,57]. A recent study conducted by Valiyambath et al. [56] demonstrated that the endophytic colonization of black pepper by Trichoderma isolates may contribute significantly to improved tolerance against both biotic and abiotic stresses (such as pathogen infection and drought stress) through mechanisms such as pathogen suppression, stimulation of plant defense responses, and promotion of plant growth.

In parallel, soil microbiome profiling has improved our understanding of the relationships between microbial diversity, soil health, and crop productivity [57,58]. Inoculation with arbuscular mycorrhizal fungi (AMF) has been shown to reduce the incidence of foot rot disease in black pepper seedlings while improving soil health and fertility [44,53]. Similarly, plant growth-promoting rhizobacteria (PGPR) have gained considerable attention as environmentally friendly biocontrol agents and biofertilizers due to their ability to enhance nutrient availability, stimulate plant growth, and improve overall crop productivity in black pepper [59]. Beneficial microbes, including Trichoderma, AMF, and PGPR, can suppress soil-borne pathogens through multiple mechanisms such as competition, parasitism, and niche exclusion, while simultaneously inducing systemic resistance and activating plant defense pathways [60,61,62]. Collectively, these microbiome-based approaches offer sustainable and environmentally friendly alternatives for improving stress resilience, disease resistance, and productivity in black pepper cultivation.

## 4. Emerging Strategies and Future Perspectives

**Figure 2:**
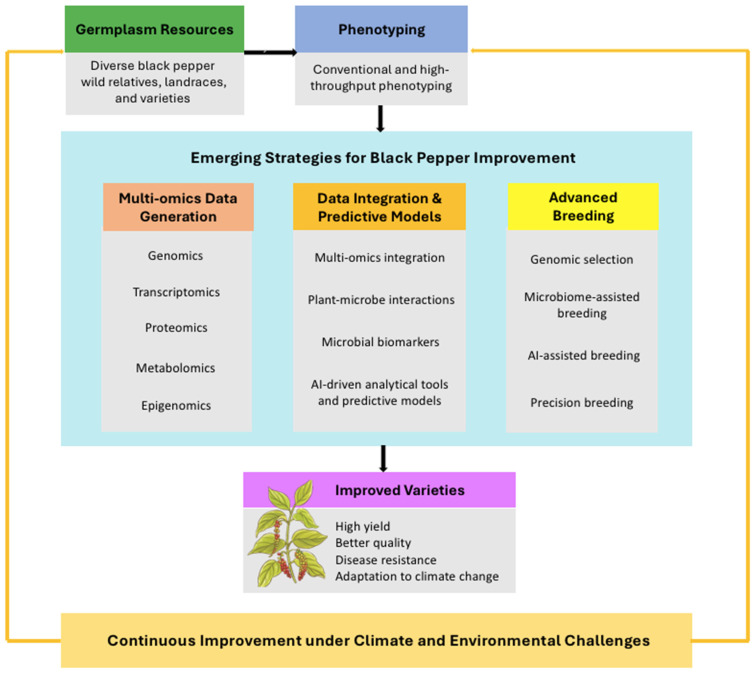
Conceptual framework for accelerating climate-resilient improvement of *P. nigrum* through the integration of germplasm resources, multi-omics, microbiome-assisted approaches, predictive modeling, and advanced breeding strategies.

Plants are frequently exposed to multiple stressors simultaneously rather than individual stresses occurring in isolation. Climate-related stresses (such as drought), soil degradation, nutrient imbalances, and pests often interact to create complex stress environments that collectively constrain crop productivity and sustainability [63]. To fully harness the potential of modern breeding technologies in black pepper, future research should establish an integrated framework that combines diverse germplasm resources, high-throughput phenotyping, multi-omics analyses, and AI-driven approaches [48,64,65]. Such a framework (Figure 2) would facilitate the systematic integration of current and emerging approaches to accelerate the development of stress-resilient cultivars. The development of high-quality pan-genomes, and ultimately super pan-genomes, encompassing cultivated varieties, landraces, and wild relatives will provide a valuable foundation for identifying novel alleles, structural variants, and candidate genes associated with stress adaptation in black pepper. Despite the availability of a reference genome containing an estimated 63,466 gene models, the functional annotation of the black pepper proteome remains largely unexplored, highlighting an important knowledge gap for future research [33].

In parallel, High-Throughput Phenotyping (HTP) platforms incorporating high-resolution sensor and imaging technologies such as thermal, hyperspectral, and multispectral sensors, as well as chlorophyll fluorescence imaging, could enable rapid screening of large germplasm collections for traits related to berry quality, photosynthetic performance, drought tolerance, and disease resistance [66]. When coupled with comprehensive phenotypic evaluation of traits such as yield, berry quality, biotic and abiotic stress tolerance, these resources will facilitate the identification of superior germplasm and accelerate marker-assisted, genomic-assisted, and AI-assisted breeding programs (Figure 2).

The increasing availability of large-scale biological datasets also creates opportunities to leverage systems biology, network analysis, and machine learning for crop improvement. AI-driven analytical tools, including Random Forest, Bayesian Networks, and deep learning models, have been successfully applied in major crops (such as rice, maize, and wheat) for trait prediction and candidate gene discovery. Similar approaches could be adapted for black pepper to integrate genomic, transcriptomic, metabolomic, phenotypic, and environmental datasets, thereby uncovering key regulatory networks, molecular biomarkers, and genomic regions associated with stress adaptation and productivity [67,68,69]. Such predictive models could also improve selection accuracy and reduce the breeding cycle.

Another promising frontier lies in deciphering the complex interactions between black pepper and its associated microbiome. Beneficial rhizosphere and endophytic microorganisms play important roles in nutrient acquisition, disease suppression, and stress adaptation, yet their interactions with host genetic and molecular networks remain poorly understood in crops, including black pepper [52,70]. Integrating microbiome profiling with plant multi-omics datasets offers an opportunity to identify microbial biomarkers, functional microbial consortia, and plant–microbe interaction networks associated with resilient phenotypes. These discoveries could pave the way for microbiome-assisted breeding, where genotypes capable of recruiting beneficial microorganisms are preferentially selected, as well as the development of precision bioinoculants containing AMF, PGPR, or *Trichoderma* strains to enhance productivity and stress resilience under field conditions [10,59,71,72].

As climate change and emerging disease pressures continue to threaten black pepper production, there is an increasing need for the continuous evaluation and refinement of improvement strategies under evolving environmental challenges (Figure 2), including drought, soil degradation, and emerging pests and diseases. By integrating emerging biological insights with breeding innovations through a continuous feedback process, black pepper improvement can transition from conventional trait-based selection towards predictive, precision, and climate-smart breeding strategies [48,73].

## 5. Conclusions

Future black pepper improvement will benefit from an integrated breeding framework that combines diverse germplasm resources, multi-omics technologies, microbiome-assisted strategies, AI-driven analytics, and modern breeding approaches supported by comprehensive phenotyping. To realize the full potential of these approaches, several research priorities should be addressed. These include the development of high-quality reference and pan-genomes representing the genetic diversity of cultivated and wild black pepper germplasm and the generation of large-scale multi-omics and microbiome datasets across diverse environmental conditions. In addition, greater emphasis should be placed on the functional validation of candidate genes, microbial taxa, and molecular pathways associated with stress resilience, as well as the integration of these datasets into predictive breeding frameworks. Addressing these priorities will accelerate the development of black pepper cultivars with improved yields, berry quality, resistance to biotic and abiotic stresses, and adaptation to changing climate conditions.

## Figures and Tables

**Table 1:** Stress-Responsive Genes and Gene Families Reported in *P. nigrum*.

Gene/Gene Family	Role(s)	Reported Significance in *P. nigrum*	Ref.
ERF TFs	Abiotic stress responses	Abiotic stress (e.g., drought) adaptation	[33]
NAC TFs	Abiotic stress responses	Abiotic stress (e.g., drought and salinity) adaptation and osmotic regulation	[33]
WRKY TFs	Abiotic stress signaling and responses	Activation/Regulation of genes associated with oxidative stress, drought, salinity, and temperature extremes	[34]
MYB TFs	Abiotic stress response and secondary metabolism regulation	Activation/Regulation of genes associated with antioxidant responses	[35,36]
Piperine biosynthesis genes	Pungency, taste, and stress adaptation	Alkaloid biosynthesis, berry pungency and taste	[37,38]
Antioxidant defense genes (i.e., SOD, APX, CAT, and POD)	ROS detoxification, hydrogen peroxide scavenging, and oxidative stress regulation	Regulation of cellular redox, protection of cells against oxidative damage under abiotic stress (e.g., drought)	[39]
*R* and *PR* genes	Pathogen effectors recognition and resistance	Putative genes for breeding against foot rot disease caused by *P. capsici*	[40,41]
*LOX* genes	Pathogen responses	Regulation of JA-mediated defense signaling	[42]

TFs—Transcription factors.

## Data Availability

No new data were created or analyzed in this study. Data sharing is not applicable to this article.
